# Pronghorn (*Antilocapra americana*) enamel phosphate δ^18^O values reflect climate seasonality: Implications for paleoclimate reconstruction

**DOI:** 10.1002/ece3.8337

**Published:** 2021-11-23

**Authors:** Danielle Fraser, Sora L. Kim, Jeffrey M. Welker, Mark T. Clementz

**Affiliations:** ^1^ Palaeobiology Canadian Museum of Nature Ottawa ON Canada; ^2^ Department of Biology Carleton University Ottawa ON Canada; ^3^ Department of Earth Sciences Carleton University Ottawa ON Canada; ^4^ Department of Paleobiology Smithsonian Institution National Museum of Natural History Washington District of Columbia USA; ^5^ Department of Geology and Geophysics University of Wyoming Laramie Wyoming USA; ^6^ Department of Life and Environmental Sciences University of California Merced California USA; ^7^ Department of Biological Sciences University of Alaska Anchorage Anchorage Alaska USA; ^8^ Department of Ecology and Genetics University of Oulu Oulu Finland; ^9^ UArctic Oulu Finland; ^10^ Program in Ecology University of Wyoming Laramie Wyoming USA

**Keywords:** oxygen stable isotopes, palaeoclimate, pronghorn, seasonality

## Abstract

Stable oxygen isotope (δ^18^O) compositions from vertebrate tooth enamel are widely used as biogeochemical proxies for paleoclimate. However, the utility of enamel oxygen isotope values for environmental reconstruction varies among species. Herein, we evaluate the use of stable oxygen isotope compositions from pronghorn (*Antilocapra americana* Gray, 1866) enamel for reconstructing paleoclimate seasonality, an elusive but important parameter for understanding past ecosystems. We serially sampled the lower third molars of recent adult pronghorn from Wyoming for δ^18^O in phosphate (δ^18^O_PO4_) and compared patterns to interpolated and measured yearly variation in environmental waters as well as from sagebrush leaves, lakes, and rivers (δ^18^O_w_). As expected, the oxygen isotope compositions of phosphate from pronghorn enamel are enriched in ^18^O relative to environmental waters. For a more direct comparison, we converted δ^18^O_w_ values into expected δ^18^O_PO4*_ values (δ^18^O_W_‐_PO4*_). Pronghorn δ^18^O_PO4_ values from tooth enamel record nearly the full amplitude of seasonal variation from Wyoming δ^18^O_W‐PO4*_ values. Furthermore, pronghorn enamel δ^18^O_PO4_ values are more similar to modeled δ^18^O_W‐PO4*_ values from plant leaf waters than meteoric waters, suggesting that they obtain much of their water from evaporated plant waters. Collectively, our findings establish that seasonality in source water is reliably reflected in pronghorn enamel, providing the basis for exploring changes in the amplitude of seasonality of ancient climates. As a preliminary test, we sampled historical pronghorn specimens (1720 ± 100 AD), which show a mean decrease (a shift to lower values) of 1–2‰ in δ^18^O_PO4_ compared to the modern specimens. They also exhibit an increase in the δ^18^O amplitude, representing an increase in seasonality. We suggest that the cooler mean annual and summer temperatures typical of the 18th century, as well as enhanced periods of drought, drove differences among the modern and historical pronghorn, further establishing pronghorn enamel as excellent sources of paleoclimate proxy data.

## Graphical abstract

We evaluate the use of stable oxygen isotope compositions (δ^18^O in phosphate; δ^18^O_PO4_) from pronghorn (*Antilocapra americana* Gray, 1866) enamel for reconstructing paleoclimate seasonality by serially sampling lower third molars of recent adults (died 1970, 1972, 2010) from Wyoming. We found that pronghorn δ^18^O_PO4_ values from tooth enamel record nearly the full amplitude of seasonal variation from Wyoming. Collectively, our findings establish that seasonality in source water is reliably reflected in pronghorn enamel, providing the basis for exploring changes in the amplitude of seasonality of ancient climates.

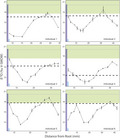

## 1 INTRODUCTION

Today, climate can be measured at a range of spatiotemporal scales through assessment of stable isotope ratios in precipitation (δ^18^O, δ^2^H; Bailey et al., 2019; Daniels et al., 2017; Liu et al., 2014; Vachon et al., 2010a; Welker, 2012). Direct measurements of precipitation stable isotope values are, however, rarely available for the reconstruction of past climates. Ice cores are a rich, high fidelity source of paleoclimate proxy data (δ^18^O, δ^2^H, δ^13^C from CO_2_ in trapped air bubbles) (Klein et al., 2016), but are limited primarily to the late Pleistocene (2.6 Ma–11.7 ka) and Holocene (11.7 ka to present; MacGregor et al., 2020; Rasmussen et al., 2006). Furthermore, they cannot approximate local climates (e.g., for mid‐latitude North America), as they often come from very spatially distant localities (e.g., Greenland; Capron et al., 2021). An array of other climate proxies have therefore been applied to paleoclimate reconstruction in deeper time, including stable isotope analysis (δ^18^O, δ^13^C) of animal hard tissues, paleosols, leaf waxes, and diatoms from lake sediments (Bailey et al., 2018; Clementz, 2012; Daniels et al., 2017; Fox et al., 2012; Green et al., 2018; Koch, 2007; Kohn & Cerling, 2002; Kohn & Dettman, 2007; Macfadden, 2000; Stevenson et al., 2010), physical properties of paleosols (i.e., depth to the calcic horizon; Cerling et al., 1989; Retallack, 2001; Sheldon & Tabor, 2009; Stevenson et al., 2010), leaf morphology (Dunn et al., 2015; Wilf, 1997), and vertebrate community structure (Eronen et al., 2010; Fortelius et al., 2002; Fraser & Theodor, 2013). These paleoclimate proxies are used to estimate mean annual temperatures, mean annual precipitation, vegetation structure, validate climate models, and contextualize studies of paleodiversity (Cullen et al., 2020; Fraser et al., 2014; Rose et al., 2011), but paleoclimate proxies vary considerably in their fidelity to climate, preservation potential, and spatiotemporal resolution (Axford et al., 2011). Choice of proxy should therefore be determined not only by availability in the fossil record but also efficacy as established by modern validation studies.

Stable oxygen isotope (δ^18^O) analysis has been commonly applied to paleoclimate reconstruction because stable oxygen isotope values of precipitation (δ^18^O_w_) are sensitive to surface temperatures, amount of rainfall, ambient humidity, and vapor transport (Dansgaard, 1964; Gat, 1980, 1996; Pederzani & Britton, 2019; Rozanski et al., 1993; Winnick et al., 2014; Yurtsever & Gat, 1981). Today, coastal and low altitude meteoric waters generally have higher δ^18^O values compared to waters from inland, high altitude regions across the United States, North America, and globally (Allen et al., 2019; Delavau et al., 2015; Dutton et al., 2002; Liu et al., 2010, 2014; Vachon et al., 2007, 2010b; Welker, 2000). Similarly, strong seasonal variations in air temperature at mid‐to‐high latitudes are correlated with lower δ^18^O values during cooler months, while higher δ^18^O values occur during warmer months (Gat, 1996; Vachon et al., 2007; Welker, 2000, 2012; Yurtsever & Gat, 1981). This seasonality of δ^18^O values often provides divergent moisture source values for estimating temporal variation in water sources used by plants (Alstad et al., 1999; Dodd et al., 1998; Jespersen et al., 2018; Welker, 2000). The δ^18^O values from meteoric waters, plants, and vertebrate tissues therefore provide a powerful means of reconstructing hydrological dynamics and climate. Seasonal and spatial patterns of δ^18^O values preserved in fossilized materials is a similarly powerful source of data on climates of the past (Koch, 2007).

The mammal fossil record is temporally and spatially extensive among terrestrial vertebrates, and their hard tissues (i.e., enamel, bone, cementum, and dentin) are readily preserved in the fossil record (Abler, 1985). Mammal hard tissues, particularly tooth enamel, are therefore widely used in terrestrial paleoclimate reconstructions (Macfadden, 2000). Stable oxygen isotope analysis of tooth enamel has been applied to nearly every major terrestrial mammal taxon including, but not limited to, “ungulates” (i.e., perissodactyls, terrestrial artiodactyls, and proboscideans), rodents, carnivores, and lagomorphs (Blumenthal et al., 2014; DeSantis et al., 2009; Feranec et al., 2010; Feranec & Macfadden, 2006; Fricke et al., 1998; Higgins & MacFadden, 2004; Larson et al., 2001; MacFadden, 1998; MacFadden & Cerling, 1994; Uno et al., 2013). These studies are informed by an array of experimental and observational studies of modern mammals that establish the degree to which δ^18^O values from their hard tissues are reflective of consumed waters (Lécuyer et al., 2010).

Mammal hard tissues contain a highly substituted form of biological hydroxyapatite (Ca_10_[PO_4_,CO3]_6_[OH,CO_3_]_2_) (Driessens & Verbeeck, 1990), which has been termed “bioapatite” and forms in isotopic equilibrium with body water (Luz et al., 1984). Though there is temperature‐dependent fractionation of ^18^O/^16^O, most mammals maintain a relatively constant body temperature. Therefore, the δ^18^O values of their bioapatite can be interpreted as reflecting the external environment (Koch, 2007); they are reflective of the oxygen isotope composition of ingested water, with varying offsets among taxa due to physiological and ecological mechanisms (Bryant & Froelich, 1995; Kohn, 1996; Levin et al., 2006; Luz et al., 1984). Among mammals, tooth enamel mineralizes progressively from the crown to the root, undergoing progressive mineral matrix formation followed by mineralization, preserving a time series of body water isotopic composition (Fricke et al., 1998; Kohn, 2004). Hypsodont or high crowned teeth, in particular, record 1–2 years of body water isotope composition and, in turn, changes in oxygen isotope composition of consumed environmental waters (Balasse, 2002; Fricke et al., 1998; Hoppe, Stoverb, et al., 2004; Kohn et al., 1998; Passey & Cerling, 2002; Zazzo et al., 2012). The pattern of mammal enamel formation, mineralization, and tooth replacement also ensures that their hard tissues can record climatic variations on yearly, monthly, and even weekly scales (Feranec et al., 2010; Hoppe, Amundson, et al., 2004; Koch, 1998; Koch et al., 1989; Larson et al., 2001).

Oxygen isotopic variation in tooth enamel is, however, not an instantaneous record of ingested water but is instead the consequence of an isotopic mass balance that integrates ingested water, water loss processes, body water residence times, and enamel formation and mineralization rates (Ayliffe & Chivas, 1990; Bryant & Froelich, 1995; Green et al., 2018; Kohn, 1996; Kohn & Cerling, 2002; Luz et al., 1984; Passey & Cerling, 2002). The spatial and temporal resolution of climate proxies preserved in mammal hard tissues therefore vary due to interspecific differences in internal (e.g., enamel mineralization rate and geometry, tooth development, and cellular processes that may lead to isotopic overprinting during mineralization; Fricke & O'Neil, 1996; Green et al., 2018; Hoppe, Stoverb, et al., 2004; Kohn, 1996; Kohn et al., 1998; Passey & Cerling, 2002; Trayler & Kohn, 2017), external (e.g., water sources, integration of the external environment through migration), and analytical factors (e.g., serial vs. bulk sampling; Ayliffe & Chivas, 1990; Kohn, 1996; Levin et al., 2006; Luz et al., 1990; Luz & Kolodny, 1985). Although physiological and chemical processes define tooth enamel formation and thus the degree to which the isotopic composition of ingested water is recorded, relatively high‐resolution changes in the external environment are detectible using intratooth sampling methods (Balasse, 2002; Fricke et al., 1998; Fricke & O'Neil, 1996; Green et al., 2018; Martin et al., 2008; Zazzo et al., 2012). Mammal species chosen as paleoclimate indicators should therefore (i) be abundant in the fossil record, (ii) have a geographic and temporal range appropriate for the scale of the study, and (iii) record environmental parameters of interest on relevant timescales (e.g., seasonal, monthly, or yearly changes in climate).

Antilocaprids (pronghorn and their extinct relatives) have a temporally and spatially rich fossil record. The historical geographic range of the only remaining species *Antilocapra americana* (Gray, 1866) is extensive, encompassing parts of northern Mexico, extending through much of the central and western United States and as far north as the Canadian provinces of Alberta and Saskatchewan (Hall & Kelson, 1959). Furthermore, their modern range is centered on the Great Basin of the intermountain western United States (Laliberte & Ripple, 2004), a region poorly covered by other extant nondomesticated bovids and cervids. Pronghorn are a desert and grassland species (Yoakum & O'Gara, 2000) that feeds primarily on shrubs (*Artemisia* sp.) and forbs (Olsen & Hansen, 1977; Smoliak, 1971). They undergo seasonal migrations of an average of 160 km, meaning that pronghorn may be ideal for reconstructing spatial patterns of climate because reasonable interpolations can be performed at this scale (O'Gara, 1978; Sawyer et al., 2005). Moreover, antilocaprids have historically persisted in the same regions from the Miocene (~25 Ma) to the present (Janis et al., 1998), which is not true of many mammalian taxa (e.g., equids, rhinocerotids, proboscideans), thus allowing for the direct comparison of isotopic profiles from individuals in the past and present. Though their current range is markedly reduced (Nelson, 1925; O'Gara & Yoakum, 2004), numerous specimens are preserved in the natural history museums of North America, providing opportunities to study their current and historical ecology.

Herein, we examine oxygen isotope ratios from serial samples of pronghorn tooth enamel from molars and ask whether enamel oxygen isotope values reflect the seasonal variation in modern meteoric water δ^18^O values. Our expectation is that oxygen isotope values from pronghorn molar enamel phosphates (δ^18^O_PO4_) reflect meteoric δ^18^O_w_ with a constant offset due to physiology. Variations from this hypothesis may reflect derivation of body water from a combination of ^18^O sources including lake and river waters as well as evaporated plant waters. If the oxygen isotope compositions of antilocaprid enamel (δ^18^O_PO4_) faithfully record the seasonal environmental variation, then antilocaprids are an untapped paleoecological resource; they may enable high resolution as well as spatially and temporally expansive seasonality reconstruction in North America for the late Cenozoic from which there is a rich reservoir of archived samples as museum specimens. Previously, δ^18^O_CO3_ values from pronghorn incisor enamel were shown to relate to humidity (Fenner & Frost, 2008), but incisors are formed over a relatively short period and thus provide little information regarding seasonal variations in water isotope values (δ^18^O_w_).

## 2 MATERIALS AND METHODS

Modern and archaeological pronghorn specimens were acquired from the University of Wyoming Anthropology museum and Department of Anthropology (Appendix I). All specimens were collected from wild populations in Wyoming during 1970–1972 and 2010 following deaths that were not related to this study. The archaeological specimens are radiocarbon dated to 1720 ± 100 AD (Frison, 1971), thus predating the rapid climate change typical of the mid to late 20th century (Jones et al., 2001; Mann et al., 1998). The lower third molar (m3) is one of the last to complete enamel mineralization and erupt in pronghorn (Dow & Wright, 1962); therefore, we included only individuals with erupted third lower molars. To recover the most complete isotopic time series, we included only individuals showing no or little wear of the m3. We also excluded individuals with abscesses or obvious abnormalities of the dentition or jaw bone. We extracted lower third molars using a Dremel diamond cutting wheel and serially sampled the enamel at ~2 mm intervals using a Dremel tool with a diamond taper point bit (part #7144). We collected 2–3 mg of powdered enamel for each serial sample. Further, we took bulk samples (~4–6 mg) of bone from the mandibular angle just posterior to third lower molar for each individual.

To analyze the oxygen isotope composition of phosphate (δ^18^O_PO4_), we weighed 1.5–2.0 mg of enamel and 3–4 mg of bone from each specimen. Preparation procedures for the modern specimens are from a combined approach based on Bassett et al. (2007) and Wiedemann‐Bidlack et al. (2008). We pretreated all samples with 300 μl of 2.5% NaOCl for approximately 20 h to remove organics. Bone samples were usually pretreated twice to ensure complete organic removal (or more if there was continued gas production). Samples were then rinsed with deionized (DI) water 5 times and dried overnight at 50°C. We then dissolved the remaining powder in 100 μl of 0.5 M HNO_3_ overnight. To neutralize the solution and precipitate CaF_2_, 75 μl of 0.5 M KOH and 200 μl of 0.36 M KF were added. Samples were centrifuged to pellet the CaF_2_, and the supernatant was transferred from the vials to reaction vessels. We precipitated silver phosphate with 250 µl of silver amine solution (0.2 M AgNO_3_, 0.35 M NH_4_NO_3_, 0.74 M NH_4_OH) plus 3–6 drops of 0.1 M AgNO_3_ to initiate the precipitation. Samples were placed in a heat block at 50°C overnight in a fume hood to allow for maximum crystal growth. The silver phosphate crystals were rinsed five times with ~2 ml of DI water to remove residual silver amine solution. After the samples dried overnight at 50°C, 200–300 μg were weighed into pressed silver capsules and stored in an oven flushed with N_2_ until isotopic analysis. Preparation of historical specimens used a rapid silver phosphate technique from Mine et al. (2017), which demonstrated δ^18^O value fidelity to the slow precipitation method in Wiedemann‐Bidlack et al. (2008). Briefly, the historical samples were similarly treated for organic removal, but hydroxyapatite was dissolved in 50 μl 2 M HNO_3_ while CaF_2_ precipitated with 30 μl 2.9 M HF and neutralized with 50 μl of 2 M NaOH. Steps to pellet and isolate CaF_2_ were similar between methods. To precipitate silver phosphate, we added 180 μl silver amine solution (0.37 M AgNO_3_ and 1.09 M NH_4_OH) and adjusted pH to 5.5–6.5 using dilute HNO_3_, which was shown to maximize phosphate recovery (Mine et al., 2017). The silver phosphate crystals were rinsed and dried similar to the modern samples as outlined above.

The δ^18^O value of silver phosphate was measured after conversion to CO in a Temperature Conversion Elemental Analyzer (TC/EA, Thermo Scientific) coupled with a Conflo IV (Thermo Scientific) to a continuous flow isotope ratio mass spectrometer (CF‐IRMS, Thermo Scientific Delta V) (modern specimens at UWSIF and historical samples at SIELO UCM). At the UWSIF, three in‐house reference materials (two silver phosphate and one benzoic acid) were used to normalize isotopic values and check the effectiveness of pyrolysis within and between runs (modern samples at UWSIF: silver phosphate [ARCOS = 10.6 ± 0.5‰, *N* = 4 per run; UWSIF‐33 = 22.1 ± 0.6‰, *N* = 6 per run] and benzoic acid [UWSIF‐21 = 24.9 ± 0.2‰]). Variation in δ^18^O values exhibited by these reference materials was <0.4‰ (one standard deviation). In addition, at the UWSIF, we monitored the potential for isotopic alteration during sample preparation by precipitating silver phosphate from a synthetic hydroxyapatite (*N* = 3, 17.5‰ 1σ < 0.3‰ per run) and NIST 120c (*N* = 3, 22.3‰ with 1σ < 0.3‰ per run). We did not use in‐house standards at SIELO UCM. All samples were analyzed in triplicate. For the modern samples, all δ^18^O_PO4_ values are reported relative to the standard V‐SMOW on a scale such that IAEA 601 (benzoic acid) and IAEA 602 (benzoic acid), respectively, are +23.3‰ and +71.4‰. For the historical samples, all δ^18^O_PO4_ values are reported relative to the standard V‐SMOW on a scale such that IAEA 601 (benzoic acid) is +23.3‰, USGS‐80 is +13.1‰, and USGS‐81 is +35.4‰.

The spacing between enamel carbonate and phosphate δ^18^O values is often used as a check for diagenesis (Koch et al., 1997). To check the carbonate phosphate isotopic spacing, we also analyzed a subset of enamel samples for carbonate δ^18^O values as per (Koch et al., 1997; Kohn & Cerling, 2002). We weighed 1 mg of enamel and 5 mg of bone for each sample analysis of δ^18^O_CO3_ values. To remove organic matter from the bone samples, we used 2%–3% H_2_O_2_ at a ratio of 1 ml per 25 mg of sample, leaving the caps of the microcentrifuge tubes open to allow the escape of gas for 24 h. We did not pretreat the enamel samples to remove organic matter due to the minimal organic content of enamel. Only the bone was pretreated for carbonate analysis. Similar to the phosphate preparation, pretreatment was repeated until gas production ceased. We rinsed the bone samples 5 times with DI water to remove H_2_O_2_ from solution. We then added 1 M CH_3_COOH with Ca acetate buffer (pH = 4.5) to remove nonlattice bound carbonates using the same ratio as the preceding step. Samples were rinsed five times with DI water, dried for 24 h in a freeze drier, and ~1 mg weighed into exetainer tubes for isotopic analysis. Once all samples and reference materials were weighed into exetainer tubes, they were dried overnight at 50°C, the headspace flushed with He, and 100–200 μl of >100% H_3_PO_4_ was added to react for 24 h (room temperature). The CO_2_ within the headspace was sampled for isotopic composition measurement using a gas bench (Thermo Scientific) coupled to a CF‐IRMS (Thermo Scientific Delta Plus) at UWSIF. Two in‐house reference materials were used to normalize isotopic values (modern samples at UWSIF: rock [UWSIF‐18 = 2.6 ± 0.1‰ δ^13^C_CO3_, −3.3 ± 0.2‰ δ^18^O_CO3_, *N* = 4 per run] and calcium carbonate [UWSIF‐06 = 2.6 ± 0.1‰ δ^13^C_CO3_, −28.9 ± 0.2‰ δ^18^O_CO3_, *N* = 4 per run]). We monitored the potential isotopic alteration during sample preparation with one laboratory bioapatite (MSW0479, ashed manatee bone, *N* = 1 per run, δ^18^O_CO3_ = −11.6‰, δ^13^C_CO3_ = −15.4‰). Carbon isotopic compositions are reported relative to VPDB scale such that NBS 18 calcite, NBS19 TS‐limestone, and LSVEC lithium carbonate, respectively, are −5.01‰, +1.95‰, and −46.6‰. Oxygen isotopic compositions are reported relative to VPDB scale such that NBS 18 calcite, NBS19 TS‐limestone, and LSVEC lithium carbonate, respectively, are −23.2‰, −2.2‰, and −26.7‰. Variation in δ^18^O_CO3_ values of these reference materials was <0.2‰. For comparison to δ^18^O_PO4_, all δ^18^O_CO3_ values were converted to VSMOW downstream.

### 2.1 Recovering primary isotope time series

Modeling is the only means by which the primary isotopic time series can be recovered without performing a study of captive animals (Green et al., 2018), even for mammal taxa with relatively fast rates of enamel mineralization and low rates of isotopic overprinting. To recover the primary isotopic input signals from our pronghorn tooth isotopic time series, we used the mathematical model from Passey and Cerling (2002) and Passey et al. (2005) as a transfer function. The Passey and Cerling (2002) model takes primary isotope series using measured δ^18^O values from body water and reconstructs tooth enamel δ^18^O values, while also accounting for time averaging due to amelogenesis and variation in enamel maturation from the crown to the root. The Passey et al. (2005) method incorporates the Passey and Cerling (2002) time‐averaging model and uses an inverse linear system to recover the input signals from enamel isotopic time series (see MATLAB code associated with Passey et al., 2005). The model terms for the Passey and Cerling (2002) and Passey et al. (2005) models are 1*_a_* and 1*_m_*, which are the length of apposition (distance along the tooth from where a new enamel layer contacts the enamel–dentine junction and the external layer of the tooth; 7 mm) and length of maturation (the length of the tooth that is mineralizing at a given time; 13.3 mm assuming an enamel maturation time of one month as in small bovids such as sheep; Zazzo et al., 2010), respectively. The *l_a_* parameter was estimated using values reported for sheep, which range from 2 mm to 12 mm, depending on whether *l_a_* was measured on the buccal, mesial, or lingual side of the tooth and crown height (Zazzo et al., 2012). We used the midpoint of these values (7 mm) as a conservative estimate of *l_a_* in pronghorn. We note, however, that pronghorn are more closely related to giraffids than bovids (Spaulding et al., 2009). Apposition lengths are, unfortunately, unknown for close relative of antilocaprids, so we also performed a series of sensitivity analyses using δ^18^O values (‰) from a single pronghorn to understand how variation in the *l_a_* parameter may affect model outputs. We used a constant sample depth (*l_s_*) of 75% of enamel thickness because we drilled through approximately 75% of the enamel when serial sampling. The Passey and Cerling (2002) and Passey et al. (2005) models were run in R version 3.5.1 (Appendices II–IV). We note that the Passey and Cerling (2002) and Passey et al. (2005) models were devised for ever‐growing teeth, which cannot perfectly approximate the growth of pronghorn cheek teeth likely only introduces small inaccuracies. However, a study in steers suggested the model is appropriate for hypsodont ungulates (Zazzo et al., 2005).

To compare pronghorn enamel δ^18^O_PO4_ values to seasonality of environmental waters, we downloaded interpolated monthly average isotope values for precipitation from central Wyoming (43°N, 107.5°W at 6700 ft of elevation) from waterisotopes.org (Bowen, 2014; Bowen et al., 2005). These interpolations for the entire United States are based in large part on Welker's USNIP (United States Network for Isotopes in Precipitation; Welker, 2000, 2012) for the years 1989–1994 and scarce IAEA GNIP (Global Network for Isotopes in Precipitation) data from a few years in the 1960s for 6 sites across the United States (Rozanski et al., 1993). Environmental water δ^18^O_w_ values were converted into predicted phosphate values (δ^18^O_MW_‐_PO4*_) using the following equation from Kohn and Cerling (2002), which is based on empirical data from mammalian enamel:

(1) $$\delta^{18}O_{\text{PO4*}}=\left(0.9\times \delta^{18}O_{W}\right)+23$$

We use Equation 1 as a means of setting up our hypothesis that pronghorn δ^18^O_PO4_ values reflect only δ^18^O_w_ and physiological offsets. Given that Equation 1 was originally derived for evaporation insensitive species (i.e., those that rely primarily on meteoric waters), underestimation relative to measured δ^18^O values can reveal whether a focal species is evaporation sensitive (Levin et al., 2006).

To validate the interpolated δ^18^O_w_ values, we used 1000+ measured δ^18^O values from precipitation at 9 sites in Wyoming (Appendix V) from USNIP for the entire USNIP record 1989–2006 (Vachon et al., 2010b; Welker, 2000, 2012). The high‐density modern precipitation network (USNIP) provides the only site, substate, regional, and continental record of actual meteoric water values that are becoming increasing valuable in revealing the range of seasonality in modern precipitation (Vachon et al., 2010a, 2010b; Vachon et al., 2007). We made comparisons between the interpolated δ^18^O_w_ values and measured values using monthly averages across all sample sites and at the site closest to the Laramie and Rawlins region (Albany site), the site closest to the area where our modern pronghorn teeth were collected. To evaluate the relative contributions of meteoric and river waters, we also extrapolated maximum and minimum δ^18^O values from river waters in Wyoming (Kendall & Coplen, 2001). As with the meteoric waters, we converted river water δ^18^O values to enamel phosphate values.

We also obtained published δ^18^O values for water in sagebrush leaves and stems, rabbitbrush leaves, and pronghorn incisor enamel (δ^18^O_CO3_) from Fenner and Frost (2008) for comparison to δ^18^O_PO4_ values from pronghorn molar enamel (this study) as well as δ^18^O_w_ values (from interpolated and measured meteoric precipitation). All plant tissues were sampled by Fenner and Frost (2008) during the months of June and July; we converted these δ^18^O values into phosphate enamel values (δ^18^O_LW_‐_PO4*_) using Equation 1. This conversion of plant leaf water values to enamel phosphate values allowed us to set up our alternative hypothesis: Pronghorn δ^18^O_PO4_ values from enamel reflect a combination of environmental water, evaporated leaf water, and physiological mechanisms. Similarity of pronghorn δ^18^O_PO4_ values to δ^18^O_MW_‐_PO4*_ values from precipitation, lakes, and rivers or δ^18^O_LW_‐_PO4*_ values should provide information on their relative inputs. δ^18^O_CO3_ values from pronghorn incisor enamel were not converted to δ^18^O_PO4_ values and are not directly compared to δ^18^O_PO4_ values from pronghorn molars (this study).

## 3 RESULTS AND DISCUSSION

### 3.1 Seasonal variation in Wyoming δ^18^O_w_ values

**FIGURE 1:**
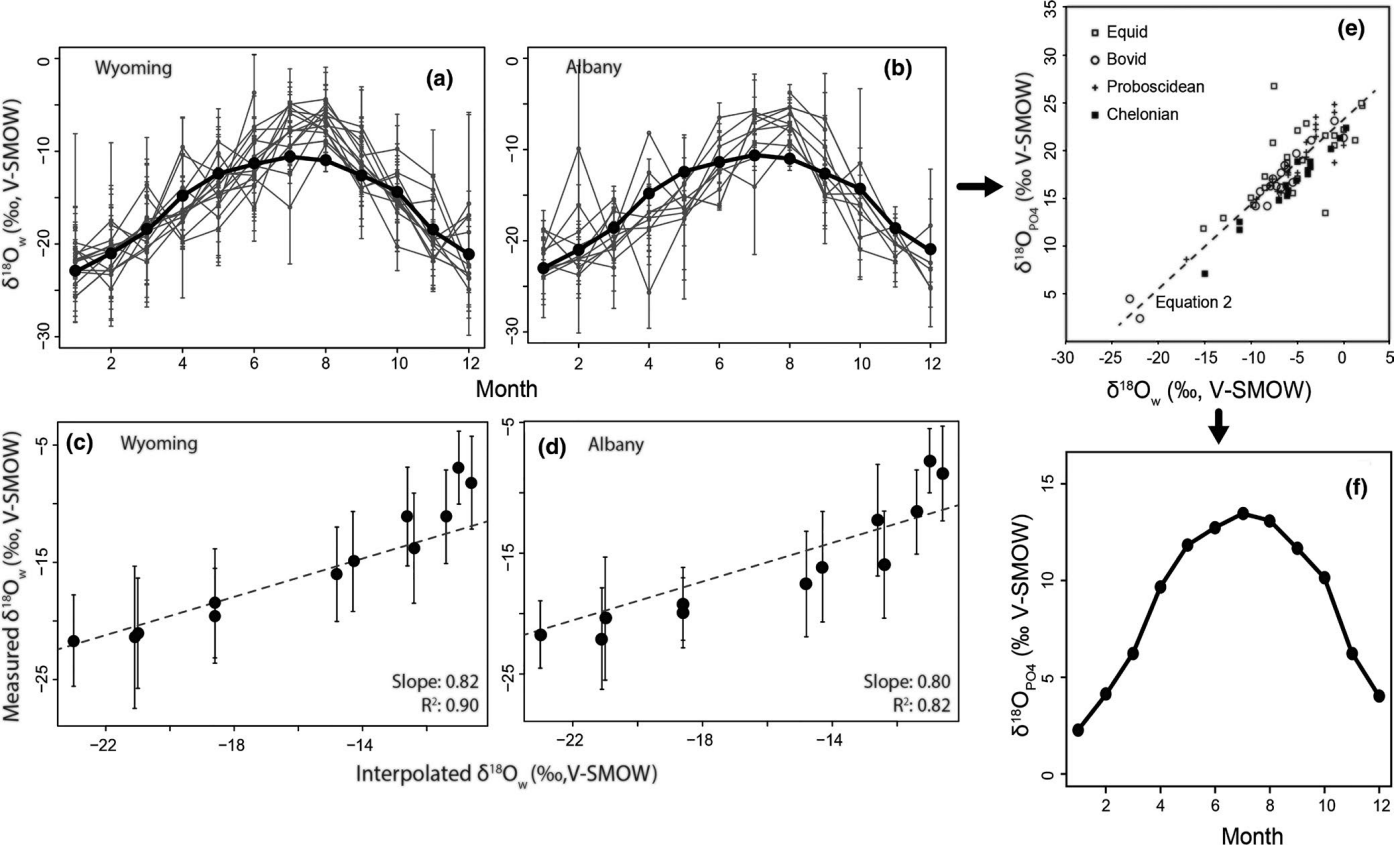
Relationship between measured (USNIP) and interpolated (waterisotopes.org) environmental δ^18^O values (‰ V‐SMOW), (a) Monthly averages of environmental δ^18^O values from 1989–2006 averaged across Wyoming (gray lines) and (b) within the Albany region (gray lines) for all sampled years plotted with interpolated average monthly values for Wyoming from waterisotopes.org (black lines), (c) correlation of measured monthly environmental δ^18^O values across Wyoming (*R* ^2^ = 0.9), (d) within the Albany region (*R* ^2^ = 0.82) with interpolated average monthly values for Wyoming from waterisotopes.org, (e) relationship between δ^18^O of drinking water and of enamel phosphate used to derive Equation 1 (modified from Kohn & Cerling, 2002), and (f) expected δ^18^O_PO4_ enamel values for a theoretical evaporation‐insensitive species

Seasonal variations in temperature and humidity are the primary factors resulting in lower δ^18^O_w_ values during cooler months and higher δ^18^O_w_ values during warmer months, a common observation in North America (Bailey et al., 2019; Vachon et al., 2007, 2010b; Welker, 2000, 2012) and Western Europe (Rozanski et al., 1992, 1993). Interpolated δ^18^O_w_ values therefore show a sinusoidal pattern, varying from −23‰ in January to −10.6‰ in July (Figure 1a, b; Allen et al., 2019; Bowen, 2014), corresponding to a modeled range of δ^18^O_MW_‐_PO4*_ enamel values of 2.3‰–13.5‰ (a range of 11.2‰; Figure 1e) for a theoretical evaporation‐insensitive species (Equation 1; Kohn & Cerling, 2002). Measured δ^18^O_w_ values from Wyoming exhibit a similar sinusoidal pattern (Figure 1a, b), but vary in amplitude among years (Figure 1a) and sites (Figure 1b). Because the interpolated δ^18^O_w_ values are based in large part on the USNIP δ^18^O_w_ data for nine Wyoming sites, they unsurprisingly fall within the range of yearly, monthly, and spatial variation of the actual measured values (note, however, that values for August tend to be low but still fall within the range of variation; Figure 1a, b; black lines). The interpolated values are thus highly correlated with the field‐measured values (Figure 1c, d; *R* ^2^ = 0.90 all Wyoming, 0.82 Albany site), although they slightly underestimate the mean of the measured USNIP δ^18^O_w_ values (Figure 1c, d; slopes <1.0).

The interpolated δ^18^O_w_ values represent an average year and smooth the effects of short‐term climate variations on actual precipitation δ^18^O_w_ values, which may be caused by climate oscillation intensities and storm track variation [e.g., El Nino; Akers et al. (2017), Liu et al. (2013), Sjostrom and Welker (2009), Welker (2012)]. In addition, Bowen and Revenaugh (2003) employ an interpolation method that reduces the estimation error of δ^18^O_w_ values by 10%–15% relative to older methods. The estimation error is improved in part by using an algorithm that includes latitude, longitude, and altitude, although this slight reduction in estimation error likely has no effect on the interpretations in this study. Due to our interest in the generalized patterns of δ^18^O_w_ seasonality in precipitation (Vachon et al., 2010b) and correspondence with variation in enamel δ^18^O_PO4_ among seasons, we compare our data to the interpolated δ^18^O_w_ (smoothed) data for the remainder of our analyses.

### 3.2 Seasonal variation in pronghorn hard tissue δ^18^O_PO4_ values

**FIGURE 2:**
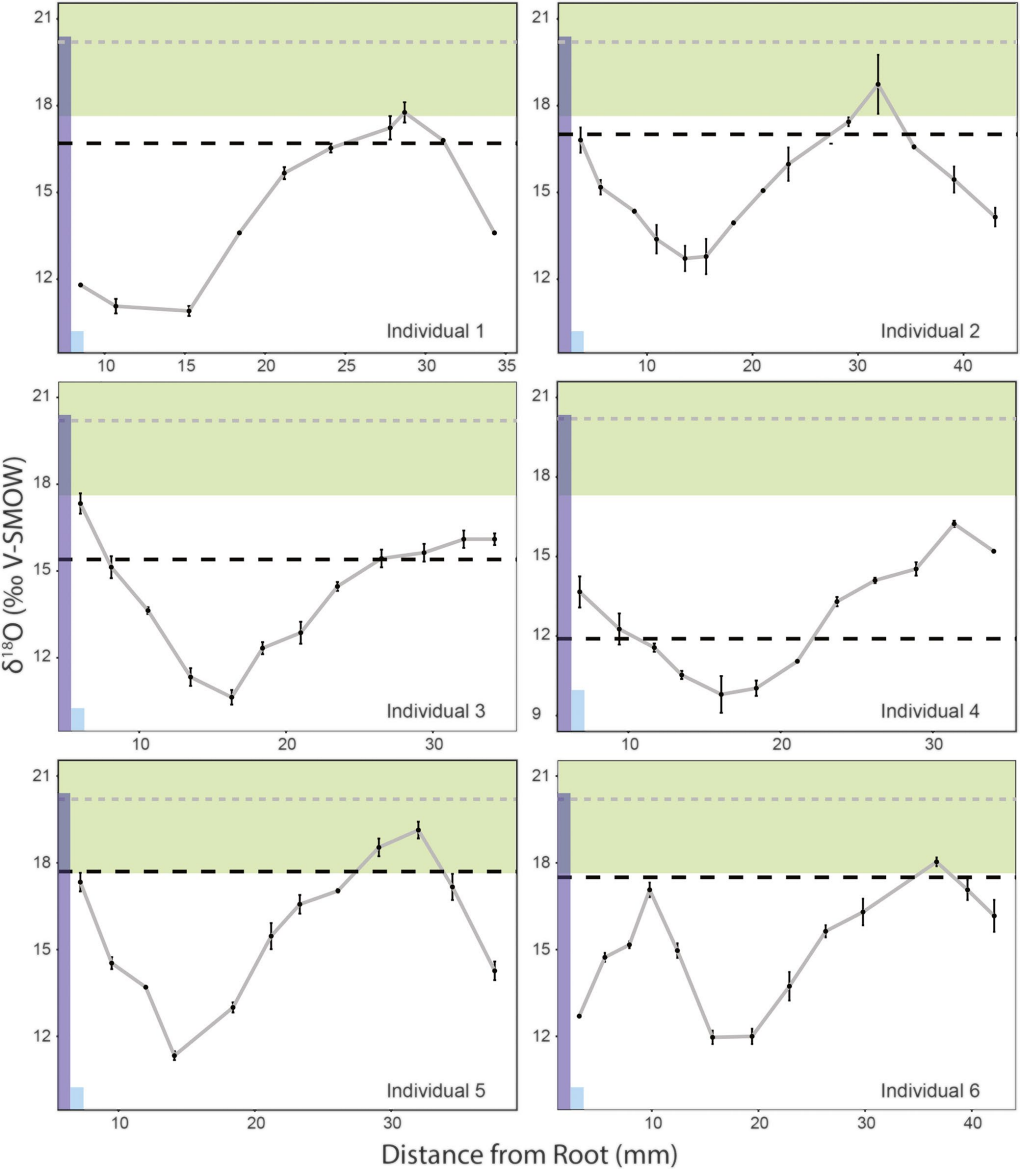
Variation in pronghorn enamel δ^18^O values (‰ V‐SMOW) for six specimens used in this study. Black dotted lines are average δ^18^O_bone_ (‰ V‐SMOW) values from the same pronghorn specimen. Gray dotted lines and green bars are predicted mean and standard deviation for δ^18^O_PO4*_ (‰ V‐SMOW) values from tooth enamel for a theoretical individual whose body water reflects only plant leaf waters during the summer months. Purple bars are predicted δ^18^O_PO4_ (‰ V‐SMOW) values from tooth enamel for a theoretical individual whose body water reflects only lake waters. Light blue bars are predicted δ^18^O_PO4_ (‰ V‐SMOW) values from tooth enamel for a theoretical individual whose body water reflects only river waters. Note that scale is different for individual 4

Though δ^18^O_CO3_ values from pronghorn incisors have been previously related to local humidity in Wyoming (Fenner & Frost, 2008), we cannot use incisors to infer seasonal variations in water isotope values (δ^18^O_w_) because they form over comparatively short periods. The cheek teeth of *Antilocapra americana* are hypsodont and the enamel of the lower third molar (m3) is laid down from occlusal surface to root from approximately 0.5 to 2 years of age (Dow & Wright, 1962). Serial samples (~10–14 samples per individual run in triplicate for 80 individual samples and 240 isotopic measurements) from the third lower molars of six modern (1970, 1972, 2010) pronghorn show similar sinusoidal patterns to seasonal variation in the interpolated δ^18^O_w_ values (Figure 2). A comparison of variation between enamel δ^18^O_PO4_ and Wyoming δ^18^O_w_ values confirms that each sampled tooth records approximately 12–18 months of the animal's life (Figure 1e), consistent with X‐ray studies, which suggest that completion of m3 mineralization occurs between 1 2/3 and 2 1/3 years of age (Dow & Wright, 1962). We suspect a small amount of wear on the teeth may have truncated the δ^18^O_PO4_ record in pronghorn enamel that we sampled. However, the teeth of all individuals clearly record at least a year of each animal's life. We therefore estimate a rate of enamel formation of ~30 mm/year for pronghorn. A value of 30 mm/year is well within the range of estimates for small bovids such as sheep, goats, and antelope (Balasse et al., 2003; Bocherens et al., 2001; Fricke et al., 1998; Fricke & O'Neil, 1996; Kohn & Cerling, 2002; Milhaud & Nezit, 1991; Suga, 1982; Witter & Míšek, 1999; Zazzo et al., 2002, 2010).

The total range for all modern Wyoming pronghorn enamel δ^18^O_PO4_ values is 9.0 to 19.9‰ (10.9‰ total range among all individuals, 5–6‰ range for each individual). The highest δ^18^O_PO4_ values from pronghorn enamel reflect the δ^18^O values of water ingested (i.e., from meteoric and/or plant sources) during the summer, while the lower δ^18^O_PO4_ values reflect water ingested during the winter. Individually, pronghorn molars record 54% to 70% of the annual range in δ^18^O values for Wyoming meteoric waters (Figure 2). The seasonal patterns in enamel δ^18^O_PO4_ values from our sample of pronghorn molars are clearly dampened. Seasonal changes in the sources of pronghorn body water as well as physiological mechanisms can lead to signal dampening and isotopic overprinting during amelogenesis and mineralization (Balasse et al., 2003; Bocherens et al., 2001; Fricke et al., 1998; Fricke & O'Neil, 1996; Kohn & Cerling, 2002; Milhaud & Nezit, 1991; Suga, 1982; Witter & Míšek, 1999; Zazzo et al., 2002, 2010). However, physiological effects can be modeled using the inverse modeling technique of Passey et al. (2005), which are used to reconstruct primary input isotopic time series (i.e., body water denoted here as δ^18^O_BW_‐_PO4*_). In our analysis, we have assumed an enamel maturation time similar to that of other extant small bovids (i.e., 3 months; Kohn, 2004). Large bovids such as cattle typically have enamel maturation times of ~6 months (Balasse, 2002; Sakae & Hirai, 1982). However, rates of enamel mineralization are considerably slower in cattle than in small bovids such as sheep and antelope (Fricke et al., 1998; Fricke & O’Neil, 1996; Kohn et al., 1998). Modeled input δ^18^O_BW_‐_PO4*_ values for all specimens in our study record 100%–120% of the total amplitude of Wyoming δ^18^O_w_ values (Figure S1), likely reflecting ^18^O‐enrichment due to evaporation from plant leaves. By serial sampling the enamel of six individuals and analyzing their δ^18^O_PO4_ values (total *N* = 80), we have resolved the full annual range of seasonal variability in δ^18^O_w_ values from Wyoming. Our sampling density and number of individuals (i.e., 6) is similar to or larger than prior studies of equids, bison, caribou, and rhinoceros teeth (i.e., 4–10 individuals) (Britton et al., 2009; Fricke & O'Neil, 1996; Hoppe, 2006; MacFadden & Higgins, 2004; Martin et al., 2008; Pellegrini et al., 2011), suggesting that large numbers of individuals are not necessarily needed to infer the total range of seasonal variation in δ^18^O_w_ values for a particular region.

In all but one case, we find that pronghorn bone δ^18^O_PO4_ values are biased toward values typical of the spring and summer, suggesting greater mineralization of bone during these months and reduction of bone remodeling during the winter months (Figure 2). Similarly, published δ^18^O_CO3_ values from Wyoming pronghorn incisors show a similar pattern to the bone. Pronghorn enamel from Fenner and Frost (2008) have a mean δ^18^O_CO3_ value of 23.6‰ (V‐SMOW; ±2.3‰ one standard deviation), thus showing bias toward values typical of spring and summer. Pronghorn bone and incisor enamel therefore appear to have limited utility for inferring seasonality but may have applications for comparing the spring and summer seasons among time periods thought to be typified by different climates. However, the rapid diagenesis of bone limits its applicability in all but Quaternary paleoecology (Koch et al., 1997). Among humans and certain other mammals, unlike the pronghorn studied herein, bone tends to be ^18^O‐depleted relative to enamel (Lee‐Thorp & van der Merwe, 1991; Webb et al., 2014). This difference may related to the locale of sampling and inherent differences in seasonality; Webb et al. (2014) sample individuals from tropical to subtropical regions, whereas the pronghorn studied herein are from the relatively high‐latitude interior of North America. Supporting our hypothesis, are data from modern equids collected from Kenya and the United States, which show that bone δ^18^O values for African horses tend to be low relative to mean for enamel, while bone δ^18^O values for North American horses tend to be higher relative to mean for enamel (Bryant et al., 1996). It is, however, outside the scope of the present paper to discern whether pronghorn bone and enamel show different offsets for individuals from different latitudes.

### 3.3 Potential sources of oxygen in pronghorn hard tissues

For comparison, we converted meteoric water δ^18^O values into δ^18^O_PO4_ values from enamel (δ^18^O_MW_‐_PO4*_) (Equation 1; Kohn & Cerling, 2002) for a theoretical evaporation‐insensitive species (Figure 1d, e). Similarity to the converted meteoric water values (δ^18^O_MW_‐_PO4*_; in amplitude and relative values) would indicate that pronghorn rely primarily on meteoric water sources and that only internal factors (i.e., ^18^O enrichment due to physiology) drive differences between enamel δ^18^O_PO4_ and δ^18^O_w_ values. However, serial enamel samples from all of the sampled modern pronghorn show ^18^O enrichment relative to δ^18^O_MW_‐_PO4*_ values of enamel for a theoretical evaporation insensitive species (Figure 2). The offset between pronghorn enamel δ^18^O_PO4_ values and δ^18^O_MW_‐_PO4*_ values varies among individuals from 4 to 9‰ (Figure 2). This result aligns with our expectation of higher δ^18^O_PO4_ values for evaporation‐sensitive species (i.e., one that derives much of its body water from evaporated sources; Levin et al., 2006). The δ^18^O_PO4_ values from bone of the same pronghorn specimens illustrate a similar pattern, showing higher δ^18^O_PO4_ values than converted environmental waters (Figure 2; dashed gray lines). Pronghorn therefore likely obtain most of their water from evaporatively ^18^O‐enriched sources, such as plants, as observed in the wild (Beale & Smith, 1970; Yakir & Sternberg., 2000), or evaporated standing water bodies (e.g., ponds and lakes) that can be found throughout the Great Basin region of the Intermountain West and other arid regions globally (Yuan et al., 2011).

The δ^18^O values from Wyoming lake waters vary from −18‰ to −2.9‰ (Henderson & Shuman, 2009), which corresponds to predicted δ^18^O_PO4*_ enamel values of 6.5‰ to 20.4‰ for a species whose body water is derived primarily from lake waters. The large variation in δ^18^O_w_ values from lake waters in Wyoming likely reflects variation in lake size and thus relative effects of evaporation. The δ^18^O values of Wyoming rivers vary between −22 and −14‰ (V‐SMOW; Kendall & Coplen, 2001), which corresponds to predicted δ^18^O_PO4*_ values of 3.2‰ to 10.4‰ (V‐SMOW) for a species whose body water is derived primarily from river waters. In Wyoming, river waters are slightly biased toward winter values, which is likely due to greater inputs by winter precipitation and snow melt, a pattern seen throughout the western United States (Dutton et al., 2002). Without prior knowledge of which lakes or rivers pronghorn are drinking from, it is difficult to determine the relative contribution of these different meteoric waters to δ^18^O_PO4_ values from pronghorn enamel. However, pronghorn enamel δ^18^O_PO4_ values are offset from river water values by similar amounts in the summer and winter months, suggesting that pronghorn do not vary the degree to which they utilize river waters throughout the year. We cannot presently discern whether δ^18^O_PO4_ values from pronghorn enamel show a consistent offset from meteoric water across their geographic range, given spatially heterogeneous δ^18^O_w_ values. A controlled feeding and drinking water trial would be a means by which this can be accomplished in the future Clemens et al. (1987). However, the consistency among the δ^18^O profiles for individuals in this study (Figure 2) suggests that pronghorn in the sampled region use similar water sources.

In semiarid regions, such as the Great Basin, water from precipitation events is rapidly lost from the soil via evaporation (Dodd et al., 1998; Welker et al., 1991) and from leaves via evapotranspiration, the magnitude of which is dependent on the amount of precipitation (Loik et al., 2004), ambient temperatures, and humidity, as well as the structural characteristics of the plants (e.g., root depths). As such, plant leaf and stem waters are ^18^O enriched relative to meteoric waters (Dongmann et al., 1974; Leffler & Caldwell, 2005; Yakir et al., 1990; Yakir & Sternberg, 2000). For example, sagebrush leaf δ^18^O values show an offset from meteoric waters of 5–8‰ during the summer months in the Great Basin (Fenner & Frost, 2008). Seasonal changes in rainfall and humidity also produce changes in plant water δ^18^O values that track the δ^18^O values of meteoric waters with larger offsets during hot, dry seasons and smaller offsets during cool, wet seasons (Dongmann et al., 1974), thus also leading to detectible seasonal variations in δ^18^O values from mammalian enamel.

We converted the oxygen isotopic composition of plant leaf waters into expected δ^18^O_PO4*_ values. We use these converted values only as a prediction for enamel that is a direct reflection of only plant water‐derived body water and as a baseline for understanding variation in δ^18^O_PO4_ values from sampled pronghorn enamel. The δ^18^O values of Wyoming sagebrush leaves have a mean value of −3.1‰ (V‐SMOW; ±2.8‰ one standard deviation) and an offset from meteoric waters of +5–8‰ during June and July (Fenner & Frost, 2008). The δ^18^O_PO4*_ values for enamel that is a direct reflection of plant leaf waters average 20.2‰ (V‐SMOW; ±2.5‰) and are 1–5‰ higher than summer δ^18^O_PO4_ values from pronghorn enamel (Fenner & Frost, 2008). Sagebrush stems have a mean δ^18^O value of −7.7‰ (V‐SMOW; ±2.1‰) and are offset from meteoric waters by ~+3–4‰ during June and July (Fenner & Frost, 2008). The δ^18^O_PO4*_ values for enamel that is a direct reflection of sagebrush stem water have a mean δ^18^O value of 16.0‰ (V‐SMOW; ±1.9‰) and are typically not ^18^O‐enriched relative to summer δ^18^O_PO4_ values from pronghorn enamel (Fenner & Frost, 2008). Rabbitbrush leaves show similar δ^18^O offsets between δ^18^O_w_ and δ^18^O_PO4_ from pronghorn enamel during the summer months (Fenner & Frost, 2008). We, thus, infer that pronghorn rely at least partially on evaporatively enriched plant leaves and stems due to the lower offset between sagebrush leaf and enamel δ^18^O_PO4_ values (compared to δ^18^O_w_ and enamel δ^18^O_PO4_ offset). Furthermore, the δ^18^O_PO4_ offset is greatest during the winter months, suggesting that pronghorn rely more heavily on evaporatively ^18^O‐enriched plant waters during the winter (~20–30 mm from tooth root; Figure 2) and more heavily on meteoric water sources during the summer. Behavioral observations also support this conclusion; pronghorn increase their reliance on plant water sources during the winter months, preferentially browsing on evergreen‐like sagebrush leaves when grasses have all senesced, and rely more heavily on meteoric waters (e.g., lakes, rivers) during the dry summer months (Dirschl, 1963). Increases in meteoric water intake in summer may be required to maintain an equitable water balance and meet greater metabolic needs when air temperatures are highest (Beale & Smith, 1970; Harvey & Welker, 2000). However, the summer is also the period when rainfall is greatest (NCDC, 2012), which should also lead to ^18^O depletion in pronghorn water sources and, thus, greater similarity of δ^18^O_w_ and enamel δ^18^O_PO4_ during the summer months (Dansgaard, 1964).

### 3.4 Inferring seasonality from pronghorn enamel

Our data suggest that δ^18^O_PO4_ values from pronghorn enamel can be used to estimate the relative amplitude of seasonal change (e.g., whether environments were more or less seasonal). We demonstrate that pronghorn enamel effectively records seasonal isotopic variation even though the enamel δ^18^O_PO4_ values reflect different balances of meteoric and vegetation water sources to body water during the winter and summer months. Ultimately, it appears plausible that the relative changes in the δ^18^O amplitude of seasonality in the past may be derived from pronghorn enamel isotope values using 6–10 individuals.

Previous studies using bison enamel to reconstruct seasonal hydrology in Wyoming do not resolve the same amplitude of seasonal variability in δ^18^O_w_ as we do using pronghorn enamel (Fricke et al., 1998). The δ^18^O_PO4_ values from Wyoming bison range from 12 to 16‰ and overlap with values from pronghorn (Figure 2b). However, the δ^18^O values show a comparatively small seasonal amplitude of 3.1‰ (figure 2B in Fricke & O'Neil, 1996), which we suggest is related to a comparatively long enamel maturation time as well as differences in drinking and migratory behavior (Kohn, 2004; Levin et al., 2006). Time averaging due to delayed enamel mineralization after amelogenesis and transgression of multiple enamel layers during sampling are two of the primary factors driving the smoothing of isotopic time series relative to the environmental water values (Passey & Cerling, 2002; Passey et al., 2005). A comparatively rapid rate of enamel mineralization in pronghorn (estimated here as ~1.5 years for amelogensis and complete mineralization) would manifest as less time averaging and higher amplitude changes in δ^18^O_PO4_ relative to commonly sampled taxa such as bison and equids (~4 years for equids), as we report herein (Fricke & O'Neil, 1996; Higgins & MacFadden, 2004; Hoppe, Stoverb, et al., 2004). Though, bison form enamel at a faster rate (~40 mm/year compared to 30 mm/year for pronghorn) than pronghorn (Higgins & MacFadden, 2004), in the absence of differences in maturation times, we would expect to find higher δ^18^O_PO4_ amplitudes from bison than pronghorn enamel, which is not what we observe. We therefore believe a shorter enamel maturation time to be one of the primary drivers of higher δ^18^O_PO4_ amplitudes as recorded in pronghorn enamel.

Pronghorn and bison also differ markedly in their drinking and migratory behaviors. Pronghorn appear to be an “evaporation sensitive” species, as evidenced by the large offset between δ^18^O_w_ and their enamel δ^18^O_PO4_ values (Figure 2), whereas bison are “evaporation insensitive” (i.e., they derive their body water from sources that are not enriched in ^18^O due to evaporation) (Levin et al., 2006). Because evaporation from plant leaves during dry, hot periods results in ^18^O‐enrichment, pronghorn capture a greater seasonal amplitude in δ^18^O_w_ than bison (Levin et al., 2006; Makarewicz & Pederzani, 2017). Pronghorn may also capture more of the seasonal variation in δ^18^O_w_ values due the fact that they continuously occupy habitats that have very strong seasonality in meteoric water δ^18^O values (O'Gara, 1978; O'Gara & Yoakum, 2004; Sawyer et al., 2005), a common pattern in the interior of all of North America, especially Wyoming, Colorado, and Nebraska (Delavau et al., 2015; Harvey & Welker, 2000; Vachon et al., 2007). In contrast, historically, bison undertook much more spatially extensive seasonal migrations, moving among more and less seasonal habitats (Larson et al., 2001). Combined, potentially more rapid enamel maturation, reliance on evaporatively enriched plant leaves for their body water, and spatially limited patterns of migration position pronghorn as excellent sources of paleoclimate proxy data. We suggest that pronghorn are excellent for reconstructing seasonality in the past relative to other large herbivores.

### 3.5 Comparison of carbonate and phosphate δ^18^O

As a preliminary check of whether the same climatic inferences can be drawn from both the δ^18^O_CO3_ and δ^18^O_PO4_ values of pronghorn enamel, we analyzed δ^18^O values of both materials from a subsample of pronghorn enamel samples. Ratios of stable oxygen isotopes from both tooth enamel carbonates (δ^18^O_CO3_) and phosphates (δ^18^O_PO4_) reflect δ^18^O_BW_ and, therefore, exogenous δ^18^O_w_ inputs in mammals (Balasse, 2002; Fricke et al., 1998; Hoppe, Stoverb, et al., 2004; Kohn et al., 1998; Passey & Cerling, 2002; Zazzo et al., 2012). Enamel δ^18^O_CO3_ and δ^18^O_PO4_ values are therefore often treated as interchangeable because they are thought to derive from the same body water source and thus the offset between them is often considered a check for diagenesis (Koch et al., 1997). Furthermore, chemical pretreatment for analysis of enamel carbonates is simpler and analyses are frequently cheaper, both important considerations for studies such as ours.

**FIGURE 3:**
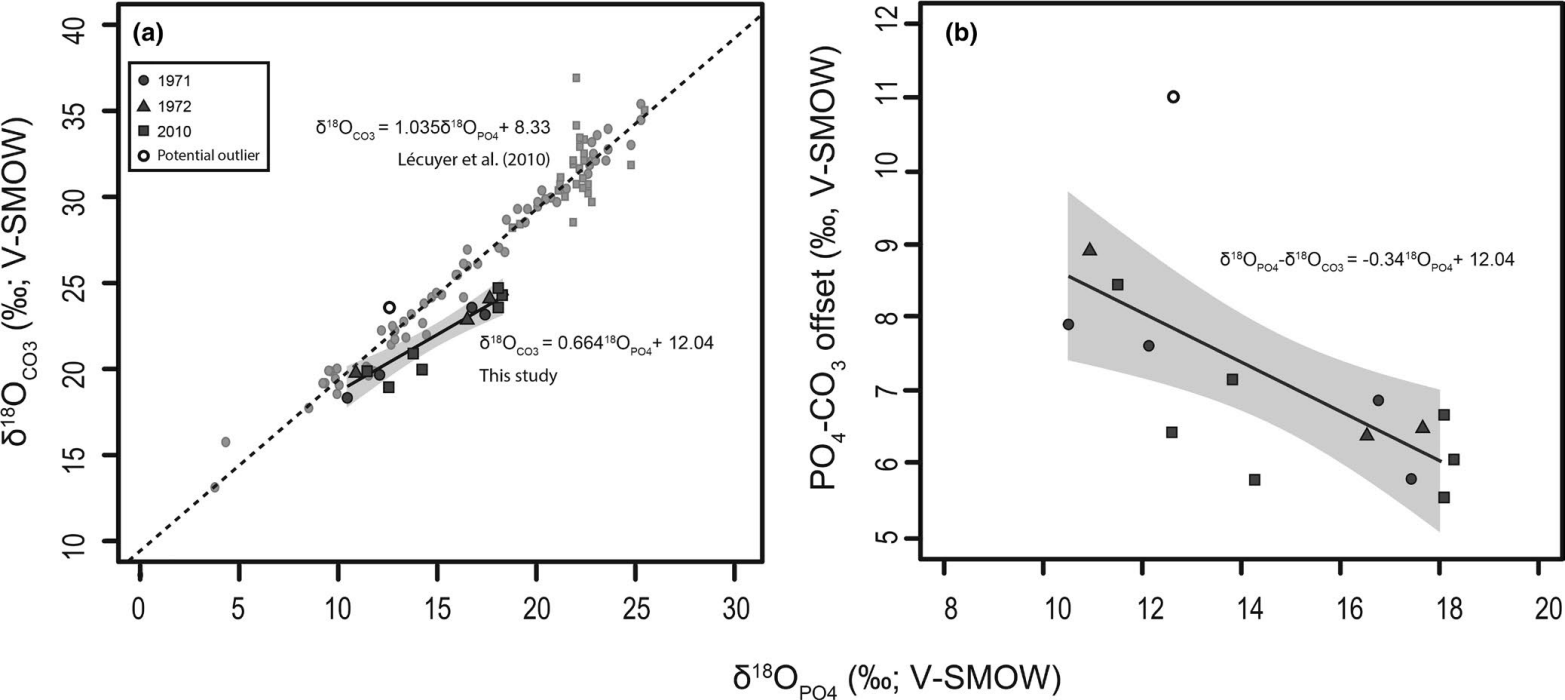
Relationship of Wyoming pronghorn enamel (a) δ^18^O_PO4_ and δ^18^O_CO3_ values (black best fit line) compared to the best fit line (dashed) derived from compilation of mammal and fish apatite (Bryant et al., 1996, Iacumin et al., 1996; Lécuyer et al., 2010; Martin et al., 2008; Shahack‐Gross et al., 1999; Vennemann et al., 2009; Zazzo et al., 2004), and (b) δ^18^O_PO4_ and carbonate phosphate δ^18^O offset showing a decline in spacing at higher precipitation temperatures

**FIGURE 4:**
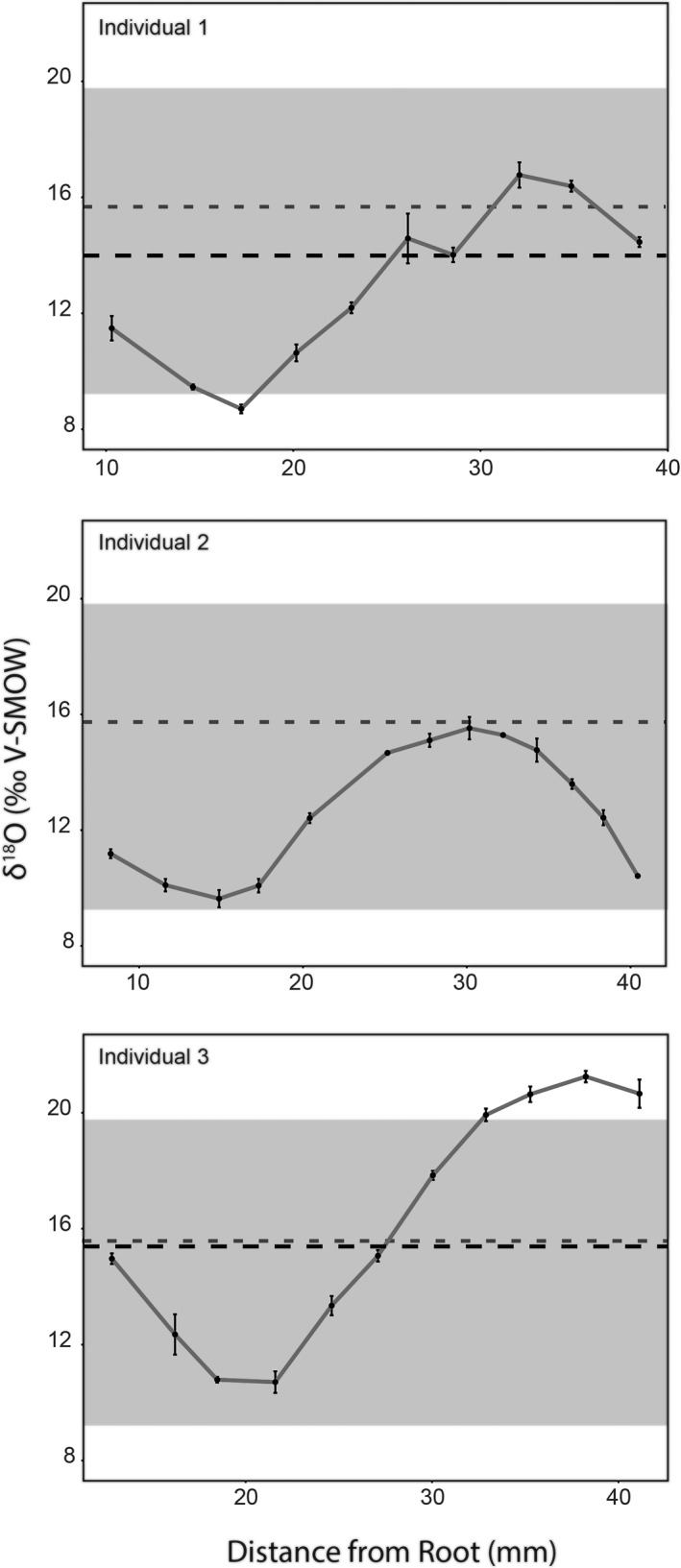
Variation in archaeological pronghorn enamel δ^18^O values (‰ V‐SMOW) for three specimens used in this study. Black dotted lines are averaged δ^18^O (‰ V‐SMOW) values from bone of the same pronghorn specimen. Bone is not available for Individual 2. Light gray shadows represent the entire (not individual) range of the modern pronghorn δ^18^O (‰ V‐SMOW) values. Gray dotted lines are the mean bone value for the modern specimens

If carbonate and phosphate oxygen are incorporated at the same time in equilibrium from the same body water pool, δ^18^O values from both compartments should show a correlation with a slope of 1.0 (Pellegrini et al., 2011). In fact, a study comparing δ^18^O_CO3_ and δ^18^O_PO4_ values from enamel shows that they record the same time periods without any spatiotemporal lag (Trayler & Kohn, 2017). Deviations from a 1:1 correlation between δ^18^O_PO4_ and δ^18^O_CO3_ values may therefore result from diagenesis or disequilibrium (Pellegrini et al., 2011). Given that we sampled individual pronghorn from the modern record, it is unlikely that diagenesis is the source of deviation from the predicted δ^18^O_CO3_: δ^18^O_PO4_ line. The methods used to prepare the samples after collection have, however, not been recorded. These might have included dermestids, low temp maceration, or boiling. In the latter case, we would expect all δ^18^O values from individual specimens to fall off the empirically derived line of best fit from Lécuyer et al. (2010), which is not what we observe (Figure 3). The offset in the majority of our samples, however, lies within the range of values reported by Lécuyer et al. (2010; Figure 4a). Several of our pronghorn δ^18^O_CO3_ and δ^18^O_PO4_ values fall below the empirically derived δ^18^O_CO3_: δ^18^O_PO4_ line (Figure 4a). We also find that the line of best fit for pronghorn enamel δ^18^O_PO4_ and δ^18^O_CO3_ values deviate from the estimated slope of 1.04 (Lécuyer et al., 2010), showing a shallower estimated slope of 0.66 ± 0.10 (Figure 3a). The variations from the predicted line appear unrelated to year of collection (Figure 3a), indicating that differences likely do not relate to date of collection or the implementation of different preparation practices in different years. Pellegrini et al. (2011) report similarly shallow lines of best fit for correlations between δ^18^O_PO4_ and δ^18^O_CO3_ from *Cervus* and *Equus* (total *N* = 8).

We show a linear decrease in the δ^18^O_CO3_: δ^18^O_PO4_ spacing (*R* ^2^ = 0.71, slope = −0.31, *p* < .001; Figure 3b), where enamel samples corresponding to summer months show less offset between δ^18^O_CO3_ and δ^18^O_PO4_ values than enamel samples corresponding to winter months (Figure 3b). We hypothesize that the apparent carbonate precipitation disequilibrium might result from temperature‐dependent effects (i.e., seasonal differences in the temperature of ingesta). That is, the ingestion of foods at a higher temperature during enamel mineralization and maturation may result in disequilibrium during phosphate and carbonate precipitation. Pronghorn also possess a carotid rete. The rete is largely de‐activated in the winter, helping to retain higher brain temperatures, but functions in water regulation and cooling during the warmer summer months (Hebert et al., 2008). To what degree the rete could influence the temperatures at which enamel is mineralized is, however, unknown. Our finding is nonetheless surprising, given that temperature‐dependent effects on δ^18^O_CO3_: δ^18^O_PO4_ spacing are expected for aquatic organisms (Lécuyer et al., 2010), but not terrestrial mammals. Our hypothesis requires further testing but we make a preliminary suggestion that δ^18^O_PO4_ values from enamel may be more appropriate for inferring seasonality using pronghorn. We also note that all of the studies reporting shallow regression lines use relatively few individuals. Thus, greater sample sizes, given the range of variation among individuals, might produce a slope closer to 1.04, as estimated by Lécuyer et al. (2010).

### 3.6 Inferences of past seasonality based on archeological specimens

As a preliminary test of our hypothesis that pronghorn tooth enamel δ^18^O_PO4_ values can be used to quantify relative changes in seasonality (i.e., combined seasonal changes in temperature, humidity, and rainfall amount) among time periods, we sampled three archaeological specimens that date to 1720 AD ± 100 years from the Eden Farson site (Frison, 1971) (34 samples run in triplicate for a total of 102 δ^18^O_PO4_ values). They show similar seasonal patterns in δ^18^O_PO4_ enamel values with variation among individuals from 21.5‰ during the putative summer months to 8.6‰ during the winter (Figure 4). Within an individual tooth, values show ranges of 8.6‰ (Individual 1), 6.6‰ (Individual 2), and 11.4‰ (Individual 3; Figure 4). Furthermore, δ^18^O_PO4_ values from enamel mineralized are 1‰ higher during the summer and 2‰ lower during the winter than for the modern specimens for two of the three individuals (Figure 4; *t* = 2.28, *p* = .02 for all samples). There is a similar offset between the bone and enamel of archaeological as among the modern specimens (Figure 4; *t* = 2.10, *p* = .05). However, bone δ^18^O_PO4_ values from the Eden Farson site are ~1.3‰ lower than for the modern pronghorn (Figure 4), excepting a single modern individual with comparatively lower bone δ^18^O_PO4_ values that shows similar values to the archaeological specimens (Figure 2). Although the differences between the modern and archaeological specimens are subtle (between 1 and 2‰) and further sampling would confirm our hypothesis, we suggest that the higher amplitude of seasonality from these archeological specimens compared to modern specimens are indicative of climate in the past 300 years. During the 17th and 18th centuries, to which the Eden Farson site is dated, Northern Hemispheric mean annual surface temperatures were as much as 0.5°C cooler than a 1961–1990 baseline (Jones et al., 2001). In Western North America, the summer months were as much as 1˚C cooler (Briffa et al., 1992). This temperature difference would translate into ~1.0‰ change in δ^18^O_w_ values (Jouzel et al., 1997). Furthermore, drought was more severe, frequent, and long‐lasting prior to 1900 (Gray et al., 2004), which would increase the magnitude of apparent δ^18^O_PO4_ seasonality from pronghorn enamel. The concurrence of past climate and seasonality between previous studies and δ^18^O_PO4_ values from archeological pronghorn provide support for our hypothesis that pronghorn enamel δ^18^O_PO4_ values can be used to infer relative changes in the amplitude of seasonality among time periods.

## 4 CONCLUSIONS

Questions concerning the interplay between terrestrial paleoclimate and diversity are being asked more frequently (e.g., whether local and regional climate impact long‐term diversity trends on land e.g., Fraser et al. (2014), Fraser et al. (2015)); therefore, paleoecologists require reliable sources of terrestrial paleoclimate data, as we unravel the processes that may contribute to the diversity of life over geologic time. We find that pronghorn enamel faithfully records the amplitude of seasonal δ^18^O_w_ value changes and suggest that isotopic time series from their enamel can be confidently used as a paleoseasonality indicator. Even without the use of the Passey et al. (2005) modeling approach, changes in the amplitude of seasonal changes can be reconstructed using relatively small sample sizes of pronghorn (and possibly their close relatives). Further, annual pronghorn spatial ranges are estimated to be approximately 100–200 km (Sawyer et al., 2005), meaning that reasonable interpolated reconstructions of paleoseasonality could be developed using pronghorn hard tissue isotopes. The δ^18^O_PO4_ values from pronghorn enamel can be averaged over areas of 100–200 km, thus reducing the demand on sampling intensity when developing Cenozoic terrestrial climate maps. Specific applications for which such paleoclimate data could be used include reconstructions of regional changes in seasonality through time, reconstruction of gradients in seasonality, and validation of terrestrial paleoclimate models. Further, we present the first preliminary evidence of possible temperature dependence in natural δ^18^O_CO3_: δ^18^O_PO4_ spacing.

## CONFLICT OF INTEREST

The authors declare no conflict of interest.

## AUTHOR CONTRIBUTIONS

**Danielle Fraser:** Conceptualization (lead); Data curation (lead); Formal analysis (lead); Funding acquisition (lead); Investigation (lead); Methodology (lead); Project administration (lead); Validation (lead); Visualization (lead); Writing‐original draft (lead); Writing‐review & editing (lead). **Sora L. Kim:** Conceptualization (supporting); Investigation (supporting); Methodology (supporting); Resources (supporting); Supervision (lead); Validation (supporting); Writing‐review & editing (supporting). **Jeffrey M. Welker:** Data curation (supporting); Funding acquisition (supporting); Resources (supporting); Writing‐review & editing (supporting). **Mark T. Clementz:** Conceptualization (supporting); Funding acquisition (supporting); Methodology (supporting); Resources (lead); Supervision (supporting); Writing‐review & editing (supporting).

## Supporting information

Fig S1

Fig S2

Fig S3

Supplementary Material

## Data Availability

The data and R code for the Passey models are available via Dryad (https://doi.org/10.6071/M3TT2J). The R code is also accessible via GitHub (https://github.com/danielleleefraser/pronghorn).
