# Supplementary figures and images for: Pronghorn (Antilocapra americana) enamel phosphate δ18O values reflect climate seasonality: Implications for paleoclimate reconstruction
Source: Ecol Evol. 2021 Nov 23;11(23):17005–21. doi: 10.1002/ece3.8337 (PMC8668790; doi:10.1002/ece3.8337)

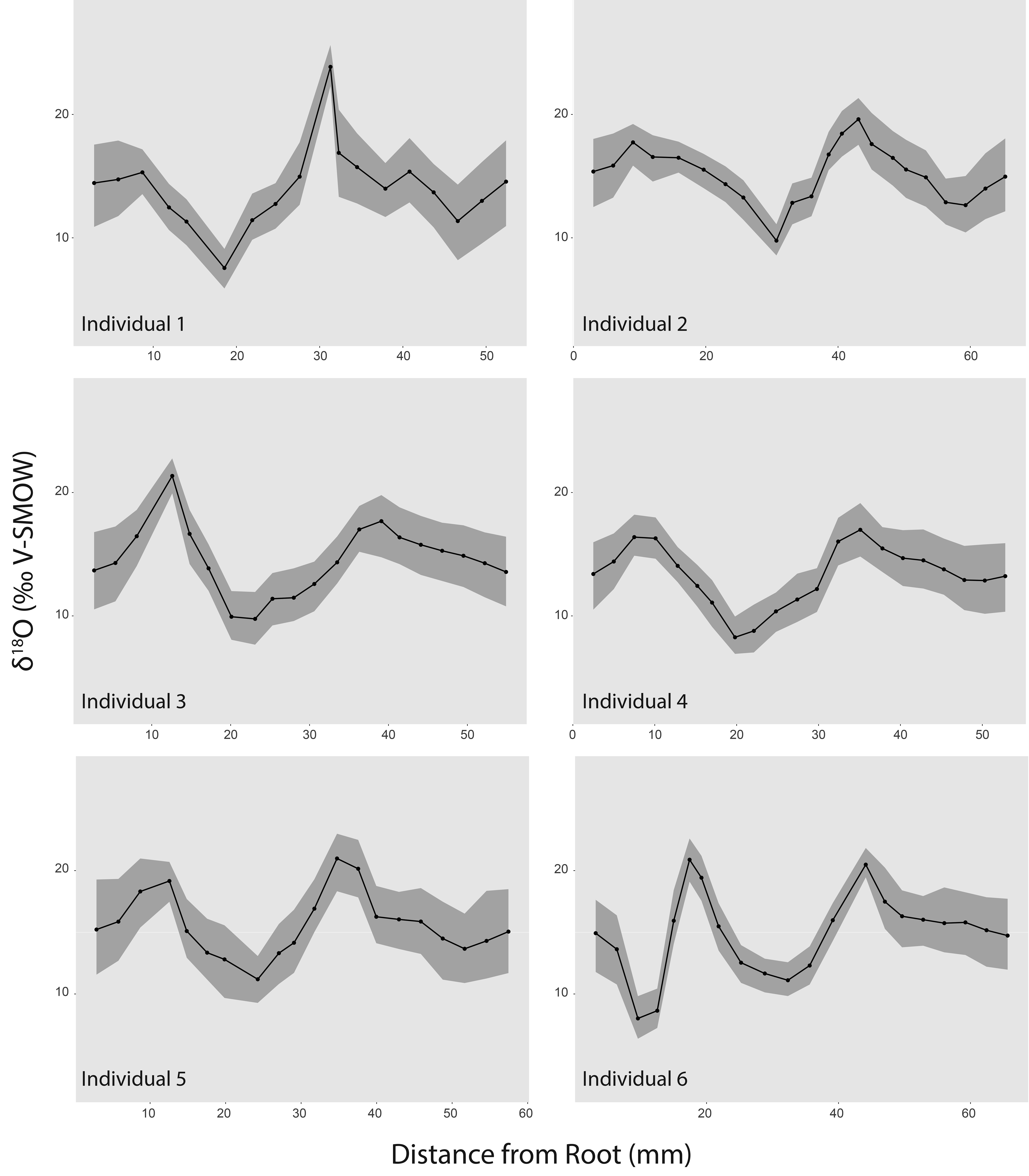

Supplement: Supplementary file 1 — Fig S1 [file ECE3-11-17005-s003.tif]

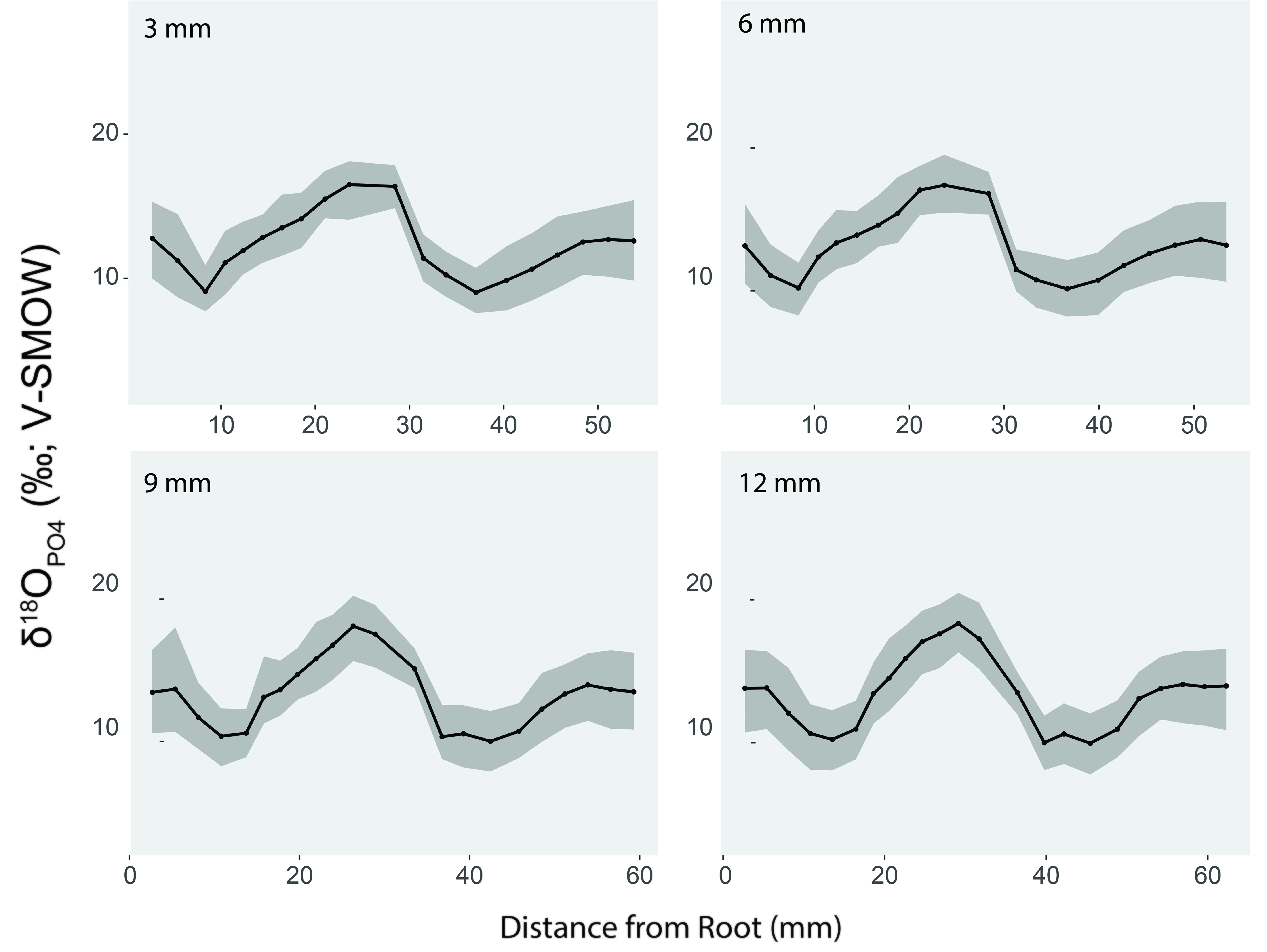

Supplement: Supplementary file 3 — Fig S3 [file ECE3-11-17005-s001.tif]
