# Supplementary material for: Pronghorn (Antilocapra americana) enamel phosphate δ18O values reflect climate seasonality: Implications for paleoclimate reconstruction
Source: Ecol Evol. 2021 Nov 23;11(23):17005–21. doi: 10.1002/ece3.8337 (PMC8668790; doi:10.1002/ece3.8337)
Supplement: Supplementary file 4 — Supplementary Material [file ECE3-11-17005-s004.docx]

FIGURE S1. Modeled input signal δ^18^O values for modern pronghorn.

FIGURE S2. Modeled input signal δ^18^O values for archaeological pronghorn.

FIGURE S3. Modeled input signal δ^18^O values for archaeological pronghorn Individual 2 with variable values for the length of apposition (la) parameter.
